# Identification of kit^M541L^ somatic mutation in chronic eosinophilic leukemia, not otherwise specified and its implication in low-dose imatinib response

**DOI:** 10.18632/oncotarget.1941

**Published:** 2014-05-01

**Authors:** Alessandra Iurlo, Umberto Gianelli, Alessandro Beghini, Orietta Spinelli, Nicola Orofino, Francesca Lazzaroni, Stefano Cambiaghi, Tamara Intermesoli, Alessandro Rambaldi, Agostino Cortelezzi

**Affiliations:** ^1^ Hematology and Transplantation Unit, Foundation IRCCS Ca' Granda Ospedale Maggiore Policlinico and University of Milan, Milan, Italy; ^2^ Oncohematology Unit of the Elderly, Foundation IRCCS Ca' Granda Ospedale Maggiore Policlinico and University of Milan, Milan, Italy; ^3^ Hematopathology Section, Division of Pathology, Department of Pathophysiology and Transplantation, University of Milan Medical School and IRCCS Ca' Granda – Maggiore Policlinico Hospital Foundation, Milan, Italy; ^4^ Department of Medical Biotechnology and Translational Medicine, University of Milan, Milan, Italy; ^5^ Hematology and Bone Marrow Transplant Unit, Azienda Ospedaliera Papa Giovanni XXIII, Bergamo, Italy; ^6^ Dermatology Unit, IRCCS Ca' Granda – Maggiore Policlinico Hospital Foundation, Milan, Italy

**Keywords:** Chronic Eosinophilic Leukemia, CEL-NOS, KIT541L, KIT mutation, Hypereosinophilic Syndrome

## Abstract

Activating mutations of KIT receptor tyrosine kinase have been reported in different neoplasms. The M541L KIT substitution (KIT^M541L^) has been described to be associated with pediatric mastocytosis, to enhance growth rate of the affected cells and to confer higher sensitivity to imatinib therapy. We investigated the presence of KIT^M541L^ in five males with chronic eosinophilic leukemia, not otherwise specified (CEL, NOS), all negative for Platelet-derived growth factor-alpha (PDGFR) or PDGFRbeta abnormalities, which responded to imatinib therapy. To assess whether the mutation was constitutive or somatic in nature, we evaluated its presence analyzing either the neoplastic or normal cell population (epidermal cells or CD3-positive T lymphocytes). KIT^M541L^ substitution was found in 4 out of 5 patients and in all it was somatic in nature. All patients were treated with low dose imatinib (100 mg daily orally), achieving complete and persistent clinical and hematological remission (median follow-up 74 months). One patient relapsed after 50 months. Our study strongly suggests to search for the KIT^M541L^ in patients with CEL, NOS, negative for PDGFRalpha and PDGFRbeta abnormalities, to identify a subgroup of cases who may benefit from low dose imatinib therapy.

## INTRODUCTION

Hypereosinophilic syndromes are a rare and heterogeneous group of disorders characterized hypereosinophilia (peripheral blood eosinophils greater than 1500/μl), lasting for longer than 6 months, and clinical manifestation related to eosinophil accumulation, either reactive or neoplastic, within different tissues. Clinical features are represented by fatigue, weight loss, pruritus, bruising, and signs of organ damage, including lung, heart, central and peripheral nervous system, and gastrointestinal tract. According to the update WHO classification (2008), the group of hypereosinophilic syndromes (HES) encompasses myeloid and lymphoid neoplasms with eosinophilia and abnormality of PDGFRalpha, PDGFRbeta, or FGFR1, chronic eosinophilic leukemia, not otherwise specified (CEL, NOS) and idiopathic hypereosinophilic syndrome (IHES) [1]. Almost all patients bearing FIP1L1/PDGFRalpha or TEL/PDGFRbeta, rearrangements respond to tyrosine kinase inhibitors (TKIs), but a striking hematologic response can also be observed in about one third of patients lacking those genetic lesions [2,3]. Recently, we described a case of hypereosinophilic syndrome with features of CEL, NOS, carrying the KIT^M541L^ variant and showing a good response to low-dose imatinib [4]. In this context an association between the KIT exon 10 variants and the clinical response of CEL, NOS to targeted treatment by imatinib was suggested.

Activating mutations of the KIT receptor tyrosine kinase, whose gene is mapped to human chromosome 4q12, have been reported in different neoplasms either hematological (e.g. acute myeloid leukemia, systemic mastocytosis) or non hematological (e.g. gastrointestinal stromal tumors, germ cell tumors, melanoma) [5-9].

Together with activating mutations, sequence variations (polymorphisms) of the KIT gene have been also described (http://www.ncbi.nlm.nih.gov/SNP/snp_ref.cgi?locusId=3815).

Among them, the M541L variant (KIT^M541L^) has been reported to be associated with pediatric mastocytosis, to heighten growth response to low levels (< 10 ng/ml) of Stem-cell Factor (SCF) of the cells carrying this substitution, and to confer higher sensitivity to imatinib [10].

On the basis of these observations we investigated the presence of the KIT^M541L^ substitution in a cohort of CEL, NOS patients who proved negative for PDGFRalpha, PDGFRbeta rearrangements, and nevertheless had showed a rapid and robust response to low dose imatinib.

## RESULTS

The main clinical and molecular features of the 5 patients analyzed in this study are summarized in table 1 and table 2. They were all male, with a median age at diagnosis of 48 years (range: 18-70 years) and diagnosed as chronic eosinophilic leukemia, not otherwise specified according to the WHO classification [1]. Cytogenetic analysis, performed on bone marrow aspirate, revealed a normal karyotype in 2 patients (#4 and #5), while the presence of chromosomal abnormalities was documented in 3 patients. In particular, in patient number 3 we identified the presence of a translocation between chromosomes 4 and 8. Fluorescence in Situ Hybridization (FISH) analysis performed on the same sample with probes for chromosome 4 and 8 suggested the involvement of q11 region on chromosome 4 and p11 region on chromosome 8. Additional experiments for breakpoint confirmation were not possible at that time.

**Table 1:** Clinical features of five cases of chronic eosinophilic leukemia, not otherwise specified (CEL, NOS)

ID	Diagnosis	Age	Gender	WBC (x10^9/L)	Eosinophils absolute count x10^9/L and (%)	HB (g/dL)	PLT (x10^9/L)	Organs Involve ments	Time to CR	Eosinophils absolute count x10^9/L and (%) after CR
1	CEL-NOS	48	M	9.8	2.1 (21%)	8.7	76	None	1 week	0,02 (0,9%)
2	CEL-NOS	60	M	13.0	1.56 (12%)	12.5	395	None	1 week	0,12 (2%)
3	CEL-NOS	18	M	6.6	2.9 (43%)	14.2	245	Gastro intestinal Tract	4 week	0,30 (6%)
4	CEL-NOS	70	M	48.9	28.8 (59%)	14.2	183	none	n.a	n.a
5	CEL-NOS	45	M	34.9	13.6 (39)	9.9	251	none	4 week	0.17 (3%)

**Table 2:** Clinical and molecular features of five cases of chronic eosinophilic leukemia, not otherwise specified (CEL, NOS)

ID	Diagnosis	Age	Gender	Cytogenetic	JAK2V 617F	BCR-ABL	FIP1L1-PDGF Ralpha	TEL-PDGFR beta	c-KIT	Imatinib response	Follow-up
1	CEL-NOS	48	M	der(5)t(1;5) (q25;34), del5(q?31;q?34)	Neg	Neg	Neg	Neg	M541L	Yes	120
2	CEL-NOS	60	M	del(5)(q32), i(17)(q10);	Neg	Neg	Neg	Neg	M541L	Yes	98
3	CEL-NOS	18	M	46,XY.ish t(4;8)(q?11;p?11) (wcp8+;wcp8+)	Neg	Neg	Neg	Neg	wt	Yes	94
4	CEL-NOS	70	M	46,XY	n.a.	Neg	Neg	n.a.	M541L	Yes	50
5	CEL-NOS	45	M	46,XY	Neg	Neg	Neg	Neg	M541L	Yes	12

Legenda: n.a.: not available; wt.: wild type

The polymerase chain reaction (PCR) was found negative for the BCR/ABL1, FIP1L1/PDGFRalpha fusion transcripts and for JAK2 mutations in all cases. TEL/PDGFRbeta was analysed in 4 out of 5 patients and resulted negative. Serum tryptase was within the normal limits or only slightly elevated, but never at the mastocytosis range (> 20 ng/ml). Bone marrow aspirates and biopsies showed a slightly to moderate hypercellular marrow, with normal or decreased erythropoiesis, sometimes with associated diserythropoietic findings. The granulopoietic series ranged from normal to slightly increased, with a constantly higher number of mature and immature eosinophils. Megakaryocytes were mature, sometimes aggregated in loose clusters, with sparse vesicular or hypolobulated nuclei. The percentage of CD34-positive hematopoietic precursors was always less than 20%. A few round mast cells (less than 5%), tryptase-positive, CD2 and CD25-negative, were sometimes evident in the interstitial space or in perivascular areas. Significant collagen fibrosis were absent in all the cases. No evidence of myeloproliferative of other hematological neoplasms was found in all the cases upon morphological and immunohistochemical evaluation.

The molecular analysis showed the presence of the KIT^M541L^ somatically acquired substitution in 4 out of 5 cases (#1, 2, 4 and 5).

Notably, we demonstrated all-heterozigous nucleotide exchanges in the 4 patients carrying this genetic variant. Moreover, this mutation was present only in the neoplastic population while it was absent in epidermal cells or T lymphocytes.

**Figure 1:**
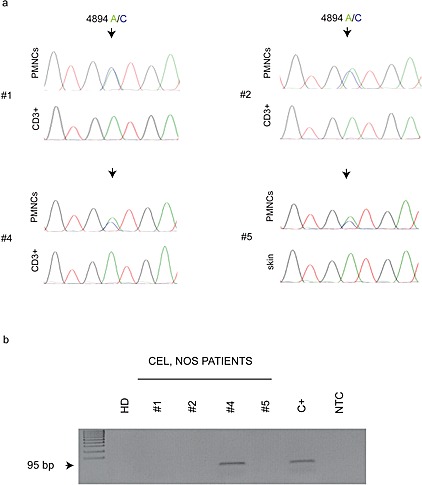
(A) Representative chromatograms of KIT exon 10 sequence on CEL, NOS patients. The analyses show the presence of the genomic variant 4894 A/C (evidenced by arrows) in the neoplastic cell population at diagnosis and its absence in the peripheral blood CD3+ T-lymphocytes or skin-derived cells. (B) ARMS-PCR assay for a sensitive detection of the KIT^M541L^ substitution in CEL, NOS patients at follow up, and BM MNCs from a healthy donor (HD). The assay output shows the persistence of the KIT^M541L^ only in the patient n. 4.

All these patients received a low-dose oral imatinib treatment (100 mg daily) and achieved a complete and persistent clinical and hematological remission. With a median follow-up of 74 months (range: 12-120 months), only one patient (numbered 4 in our series) relapsed after 50 months. No adverse events were observed during imatinib therapy in all the patients. The quality of the molecular response was checked during the follow up by a direct KIT exon 10 sequencing which was performed on genomic DNA obtained from the four CEL, NOS mutated patients (Fig. 1A). Within the limited sensitivity of this assay (which may detect the mutation in no less than 10% of the analyzed cells) the KIT^M541L^ was not detectable in all the CEL, NOS patients (data not shown). When using a higher sensitive approach (the ARMS assay, which may detect this allele variant with a sensitivity close to 3%) persistent cells harbouring KIT^M541L^ allele variant occurred only in one patient (#4), (Fig. 1B).

## DISCUSSION

In this study we found the presence of a KIT^M541L^ allele variant in four out of five CEL, NOS patients. We were able to demonstrate the somatic nature of this nucleotide substitution which was present only in the neoplastic population, while it was absent in epidermal cells or T lymphocytes. In addition, we found that imatinib, which is effective in FIP1L1-PDGFRalpha rearrangement positive hypereosinophilic syndromes [11], was highly effective also in cases bearing this KIT^M541L^ variant. Finally these results have been observed using a low dose imatinib as in FIP1L1-PDGFRalpha positive syndromes.

The intracellular tyrosine kinase activity of the transmembrane receptor KIT (CD117), is stimulated after binding of the ligand Stem cell Factor (SCF) to extracellular domain. KIT is expressed in hematopoietic progenitor cells, mast cells, interstitial cells of Cajal, germ cells and melanocytes. Activating mutations of this gene are considered to play a key role in the pathogenesis of various myeloid neoplasms (including acute myeloid leukemia and mastocytosis), characterized by increasing eosinophil precursors that are part of the malignant clone, but have not been found so far in primary hypereosinophilic syndromes [12].

The KIT^M541L^ has been described as a polymorphic variant with allele frequencies at heterozygous state close to 20%. It has been suggested that the constitutive expression of KIT^M541L^ variant confers a higher sensitivity to imatinib in pediatric mastocytosis [10] and in aggressive fibromatosis [13]. Furthermore, it was demonstrated the presence of the KIT^M541L^ variant in cell line of Merkel-cell carcinoma (MCC) and an autocrine stimulatory loop was suggested between SCF and KIT in a MCC-1 cell line which carries the M541L sequence variation [14].

Notably, we were able to demonstrate the somatic nature of this substitution and that low-dose imatinib was highly effective in cases of CEL, NOS bearing it. These findings could explain the optimal and durable responsiveness to imatinib treatment frequently reported also in patients lacking any known TKI-sensitive genetic lesions. Although this sequence variation has not been suggested as a disease-causing mutation, the high frequency of clinical association and the somatic nature of the variants, suggest to reconsider the overall role of KIT receptor and its variants in the evolution of chronic eosinophilic leukemia.

Our data therefore strongly suggest to search at diagnosis for the KIT^M541L^ in all the patients with hyperheosinophilic syndrome negative for PDGFRalpha, PDGFRbeta abnormalities, to identify an additional subgroup of patients that may benefit from imatinib therapy. Notably, our findings provide evidence that all of the four treated patients positive for KIT^M541L^ displayed a good response to low dose imatinib, supporting and extending the relevant observations that juxtamembrane KIT mutants display enhanced sensitivity to the kinase-inhibitory drug imatinib [15,16].

We are aware that our observations are limited due to the low number of the investigated patients and the good response to imatinib therapy obtained also for the single patients without the KIT^M541L^ mutation. For these reasons and considering that CEL, NOS is a rare disorders, further studies with a higher number of patients are necessary to estimate the real frequency of this mutation and the impact of the imatinib therapy.

## METHODS

We retrospectively collected data from five patients, fulfilling the criteria for CEL, NOS according to the updated WHO classification [1], followed between January 2003 and December 2012 at two Italian Hematological Departments (IRCCS Ca' Granda – Maggiore Policlinico Hospital Foundation, Milan and Azienda Ospedaliera Papa Giovanni XXIII, Bergamo, Italy) which responded to low-dose imatinib. These patients were submitted to an extensive cytogenetic and molecular analysis including BCR/ABL1, TEL/PDGFRbeta, FIP1L1/PDGFRalpha translocations and JAK2 mutations. Moreover, c-KIT analysis on exons 8, 9, 10, 11, 13, 14, and 17 was also performed. Bone marrow aspirates and biopsies were collected from all of these patients.

### Cell separation and molecular analyses

Total nucleated cells (TNC) were collected from peripheral blood or bone marrow by Hetasep (StemCell) sedimentation. CD3 positive cells were purified from whole peripheral blood by immuno-magnetic selection using a commercial kit on a semi-automatic instruments (Robosep, Stem Cell Technologies Inc. Vancouver, Canada). Total RNA was extracted using a column based system (Qiagen, Hilden, Germany) and analyzed for the presence of FIP1L1/PDGFRalpha [11], TEL/PDGFRbeta [17] and BCR/ABL1 [18] fusion transcripts as previously described. Genomic DNA was obtained by a salting out method (Qiagen, Hilden, Germany) following the manufacturer instructions. The presence of the JAK2V617F mutation was studied on DNA samples by allele specific PCR [19] and mutations in JAK2 exon 12 was explored by PCR amplification of the whole exon 12 followed by Sanger sequencing [20].

### KIT mutation analysis

Isolation of genome DNA from polymorphonuclear blood cells was performed using DNAzol reagent, according to the manufacturer's instruction (Invitrogen, San Paolo, Brazil).

The PCR amplification of c-KIT exons (8, 9, 10, 11, 17) was performed as previously described [12]. DNA sequencing of purified PCR products was carried out on an ABI PRISM 3130 XL Genetic Analyzer (Applied Biosystems, Darmstadt, Germany). Cycle sequencing was conducted using 20 ng of purified PCR fragments. Cycling conditions were as follows: 96°C for 1 min; 25 cycles at 96°C for 10 sec, 50°C for 5 sec; 60°C for 4 min. The results were analyzed using the ChromasPro software (version 1.41 Technelysium).

In order to assess whether KIT^M541L^ substitution was constitutive or somatic in nature, we investigated its presence in the 4 mutated patients (numbered 1, 2, 4, 5 in our series) also in the epidermal cells obtained from healthy skin by punch biopsies (patients number 5), or in CD3-positive T lymphocytes (patients 1, 2, and 4). All the patients gave their informed consent.

### Amplification Refractory Mutation System (ARMS) for KIT^M541L^ during the follow-up

The PCR method known as Amplification Refractory Mutation System (ARMS) [21] was carried out on genomic DNA obtained from CEL, NOS patients at follow up. The target DNA (95 bp product) was amplified using a couple of forward primers specific for the KIT 4894/C (5'-ATGATGTGCATTATTGTGC-3') genomic variant or for the 4894/A (5'-ATGATGTGCATTATTGTGA-3') normal allele respectively, and a common reverse primer (5'-TGTCAAGCAGAGAATGGGT-3').

The amplification on the thermal cycler was carried out consisting of 28 cycles: each cycle consisted of denaturation at 94°C for 30 sec, primer annealing at 64°C for 15 sec and extension at 72°C for 20 sec. In the final cycle the extension was prolonged for another 3 minutes. The PCR products was visualized after electrophoresis on a 2% agarose gel.
